# Association of *F. alocis* and *D. pneumosintes* with Periodontitis Disease Severity and Red Complex Bacteria

**DOI:** 10.3390/dj12040105

**Published:** 2024-04-12

**Authors:** Hawaabi F. M. Shaikh, Pratima U. Oswal, Manohar Suresh Kugaji, Sandeep S. Katti, Kishore Gajanan Bhat, Eswar Kandaswamy, Vinayak M. Joshi

**Affiliations:** 1 Department of Periodontology, Maratha Mandal’s Nathajirao G. Halgekar Institute of Dental Sciences & Research Centre, Belagavi 590019, India; hawaabishaikh@gmail.com (H.F.M.S.); pratima0257@gmail.com (P.U.O.); zorb@rediffmail.com (S.S.K.); 2 Centre for Advanced Medical Research, BLDE Deemed to be University, Vijayapura 586103, India; 3 Arihant Superspecialty Hospital, Belagavi 590010, India; drkgbhat@yahoo.com; 4 Department of Periodontics, School of Dentistry, Louisiana State University Health Sciences Center, New Orleans, LA 70119, USA; ekanda@lsuhsc.edu

**Keywords:** attachment loss, biofilm, dental plaque, periodontitis

## Abstract

Oral biofilms are considered the principal etiological agent in the development of periodontitis. Novel species that may contribute to periodontitis and dysbiosis have been identified recently. The study aims to evaluate the presence of *F. alocis* and *D. pneumosintes* in healthy and diseased patients and their association with clinical parameters and with red complex bacteria. The study included 60 subjects, with 30 patients each in the healthy and periodontitis groups. The clinical parameters were noted, and samples were subjected to DNA extraction followed by a polymerase chain reaction. Statistical analysis was performed using the Graph Pad Prism software. Results: *F. alocis* and *D. pneumosintes* were detected at a significantly higher percentage in the periodontitis group compared to the healthy group (*p* < 0.05). *D. pneumosintes* was significantly associated with *T. forsythia* in the periodontitis group (*p* < 0.05). Both of these organisms were present in sites with higher clinical attachment loss (*p* < 0.05). This study demonstrated that both *F. alocis* and *D. pneumosintes* were detected at a significantly higher percentage in periodontitis subjects and were detected more frequently in sites with a greater clinical attachment loss. It was also evident that both *F. alocis* and *D. pneumosintes* can be present independently of other putative periodontal pathogens.

## 1. Introduction

Microbial biofilms are considered to be the primary etiological agent for the initiation and progression of periodontitis, among other multiple contributing factors [1]. The biofilm is composed of both health-associated and pathogenic microorganisms [2,3,4]. Dental plaque is a biofilm that forms over the teeth, gingiva, and mucosa and can be composed of bacteria, fungi, and algae [5,6]. Socransky et al. elucidated that the bacteria in dental plaque form different complexes, of which the red complex bacteria consisting of *Porphyromonas gingivalis* (*P. gingivalis*), *Treponema denticola* (*T. denticola*), and *Tannerella forsythia* (*T. forsythia*) along with the orange complex bacteria are considered to be the most putative periodontal pathogens [7]. Currently, open-ended microbial identification techniques and next-generation sequencing techniques have greatly increased the understanding of microbial diversity and newer species existing in dental plaque [8,9]. This knowledge has helped us to understand the microorganisms that are involved in the dysbiosis that occurs as part of periodontal disease.

Paster et al., in 2001, pointed out the presence of novel species that could play a part in dysbiosis and periodontitis [10]. Recent studies and systematic reviews have found further proof of these new species and their putative role in periodontal pathogenesis [8,9,11]. Some of the newly discovered pathogens belong to the phyla *Bacteroidetes*, *Firmicutes*, *Proteobacteria*, *Spirochaetes*, *Synergistetes*, and *Candidatus saccharibacteria* [11]. According to a cross-sectional study, *Filifactor alocis* (*F. alocis*) was identified as one of the novel species (among the 39 other species) showing strong evidence as a periodontal pathogen [9]. *F. alocis* has unique virulence properties, which are evidenced by factors like extracellular vesicles, lipoteichoic acid, oxidative stress resistance, and protease secretion, which together trigger a pro-inflammatory cytokine response from the periodontal tissues [12,13,14]. *F. alocis* and *P. gingivalis* have been demonstrated to co-exist symbiotically, and this association intensifies the virulence properties of *F. alocis* [15,16,17]. *F. alocis* also has the ability to invade epithelial cells, similarly to *P. gingivalis*, using filapodial projections or vesicle-mediated internalization [16,17] and has been found in individuals with different grades of periodontitis [18]. *F. alocis*, being an obligate anaerobe, prefers to colonize deeper sites and is often found in the middle third and apical third of the pockets [19], which provide an ideal anaerobic environment [20]. Similar to *F. alocis*, *Dialister pneumosintes* (*D. pneumosintes*) is an obligate anaerobic bacterium that was first isolated from the nasopharynx during the flu epidemic [21]. *D. pneumosintes* has been isolated from the oral biofilm and is known to cause gingivitis, periodontitis, and other local infections [22,23,24,25,26]. Among the virulence characteristics, lipopolysaccharides in the cell wall of *D. pneumosintes* may be the most important as they trigger the release of proinflammatory cytokines and matrix metalloproteinases, leading to periodontal tissue and alveolar bone destruction [27]. The relationship between *D. pneumosintes* occurrence and the development of oral disease, its detection in young males and patients with severe periodontitis, its correlation with the pocket depth, clinical attachment loss, and active disease sites, suggests its role in the etiopathogenesis of periodontal disease [22,23,24,25,28]. *D. pneumosintes*’s association with other putative periodontal pathogens, such as *Aggregatibacter actinomycetemcomitans* and *P. gingivalis*, has also been correlated with the prevalence of severe periodontitis [29]. Recent genomic studies have increased our understanding of oral microbiota, emphasizing the need to investigate novel periodontal pathogens [9]. To the best of our understanding, this is the first study to establish the association of *F. alocis* and *D. pneumosintes* with red complex bacteria and with periodontitis disease severity in the Indian population.

The current study primarily aims to evaluate the presence of *F. alocis* and *D. pneumosintes* in periodontal health and disease, and its correlation with clinical parameters. The secondary aim is to evaluate if these novel species and the red complex bacteria are more frequently detected simultaneously or can exist independently in moderate to severe periodontitis.

## 2. Materials and Methods

The initial screening of this cross-sectional study consisted of one hundred eighty-five subjects aged between 18 and 70 years conducted between January 2016 and January 2017. The study group consisted of 87 subjects with a suspected diagnosis of moderate to severe periodontitis and 98 subjects periodontally healthy from the outpatient department. The institutional ethics review board approved the study (Certificate number: 2015-16/1118). All study participants were interviewed according to a standardized protocol, and a written informed consent was obtained from all subjects before the examination. All selected subjects who met the inclusion–exclusion criteria were divided into a healthy group (H group), consisting of subjects with a healthy periodontium, and a periodontitis group (P group) based on the periodontal parameters [27].

**Figure 1:**
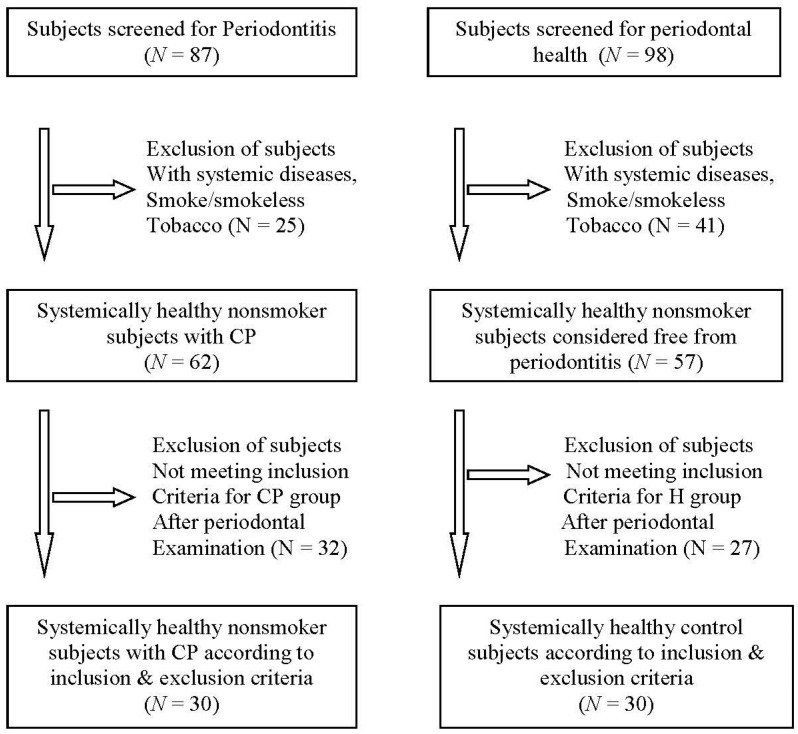
Flow diagram showing the selection of patients with periodontitis (P group) and healthy periodontium (H group), according to the inclusion and exclusion criteria.

The sample size was estimated based on a previous paper [19] with a significance level of *p* = 0.05 and a power of 95% using a conservative two-tailed testing approach. The power analysis was accomplished using G*Power 3.1 [30]. Subjects were excluded from the study if they underwent periodontal therapy or had antimicrobial therapy in the previous 3 months, had a history of any systemic diseases/conditions, were pregnant and lactating women, and smoked or consumed smokeless tobacco. The subjects who matched the inclusion–exclusion criteria were included in the study (Figure 1). This manuscript was prepared according to the STROBE cross-sectional study checklist [31] (Supplement File).

### 2.1. Clinical Examination and Subgingival Plaque Sample Collection

A comprehensive periodontal examination was carried out, and the clinical parameters recorded included plaque index (PI) [32], gingival index (GI) [33], bleeding index (BI) [34], PD, and CAL. The inclusion criteria for the H group were subjects with minimal signs of gingival inflammation or bleeding on probing (BOP) (BOP < 10%), absence of clinical attachment loss (CAL), and probing depth (PD) ≤ 3 mm. The P group included subjects with gingival inflammation with the presence of BOP, PD ≥ 5 mm, and CAL ≥ 3 mm (moderate to severe periodontitis). All participants needed to have a minimum of 20 teeth. Subgingival plaque samples were collected under strict asepsis using a sterile Gracey curette. In the P group, plaque samples were harvested from the three deepest sites with a probing depth of ≥5 mm, and in the H group, plaque samples were collected from the normal healthy gingival sulcus. The selected sites were isolated with sterile cotton rolls and air-dried, and supragingival plaque and calculus were removed. The plaque sample was then transferred into a 2 mL plastic vial containing the transport medium Tris-EDTA buffer (T.E.) and was then processed for DNA extraction. A single examiner did the clinical examination and sample collection.

### 2.2. DNA Extraction and Polymerase Chain Reaction

The DNA extraction for the plaque samples was carried out using a modified proteinase K method, as previously described [35]. Following the DNA extraction, a polymerase chain reaction (PCR) was carried out using the Ampliqon RED 2X master mix and specific primers to identify *F. alocis*, *D. pneumosintes*, *P. gingivalis*, *T. denticola*, and *T. forsythia*. Two multiplex PCR reactions were performed. One contained 16S rRNA primers specific for *F. alocis* and *D. pneumosintes* [36]. The other PCR reaction utilized 16S rRNA primers specific for *P. gingivalis*, *T. denticola*, and *T. forsythia* [37]. The primer sequences are presented in Table 1.

The PCR cycling conditions for the amplification of *D. pneumosintes* and *F. alocis* included an initial denaturation step at 95 °C for 5 min, followed by 36 cycles of a denaturation step at 94 °C for 30 s, primer annealing step at 55 °C for 1 min, and extension at 72 °C for 2 min. The thermal cycling conditions for the amplification of red complex bacteria encompassed an initial denaturation step at 95 °C for 5 min followed by 40 cycles of a denaturation step at 94 °C, primer annealing at 60 °C for 1 min, and an extension at 72 °C for 1 min. The final extension step was performed at 72 °C for 10 min.

The amplified products were separated on 2% agarose gel and subjected to electrophoresis in 1X Tris-Acetate EDTA buffer. The gel was stained using 0.5 µg/mL ethidium bromide and visualized using a Gel documentation system (Major Science, Saratoga, CA, USA). A 100 bp DNA ladder simultaneously loaded on the gel was used as a marker. The specific band size corresponding to each bacterium was identified and recorded as positive amplification.

### 2.3. Statistical Analysis

The demographic data and clinical parameters (GI, PI, BI, PD, and CAL) were compared using chi-squared test, Student’s *t*-test, and Mann–Whitney U test. The detection of *F. alocis*, *D. pneumosintes* and red complex bacteria in each group and the association of red complex bacteria with the presence of *F. alocis* and *D. pneumosintes* were analyzed using a Fisher’s exact test. The association of *F. alocis* and *D. pneumosintes* with CAL was analyzed using the Freeman–Halton extension of the Fisher’s exact test. Any *p*-value ≤ 0.05 was considered to be statistically significant. All calculations were performed using the Graph Pad Prism software (Version 5; GraphPad Software Inc., La Jolla, CA, USA).

## 3. Results

This current study consisted of 60 participants, with 30 individuals each in the H group and the P group. The H group consisted of 18 females and 12 males, with a mean age of 44.03 ± 10.11 years, and the P group consisted of 17 females and 13 males, with a mean age of 42.07 ± 10.60 years, with no statistical difference seen between the groups. All other clinical parameters recorded showed statistical differences between the H group and the P group (Table 2).

The comparison of the detection frequency of *F. alocis*, *D. pneumosintes*, and the red complex bacteria between the healthy and P groups was performed. *F. alocis* was detected in 20% of samples in the H group and 53.3% of samples in the P group, and the comparison was statistically significant (*p* < 0.05) (Table 3). The detection of *D. pneumosintes* was 20% and 66.67% in H and P groups, respectively, and the difference was significant (*p* < 0.05) (Table 3). Among the red complex bacteria, *T. denticola* was detected in 20% and 53.3% in the H and P groups, and the difference was significant (*p* < 0.05), while the detection of *P. gingivalis* and *T. forsythia* between the groups was non-significant (Table 3).

The presence of F. alocis was not significantly associated with the presence of any red complex bacteria (Table 4). However, *D. pneumosintes* showed a significant association with the co-presence of *T. forsythia* in the P group (*p* < 0.05) (Table 5). Both *P. gingivalis* and *T. denticola* did not show any association with the presence of *D. pneumosintes* (Table 5). The presence of *F. alocis* and *D. pneumosintes* at different clinical attachment loss levels showed that both of these organisms were detected at a higher frequency in sites with a greater attachment loss (*p* < 0.05) (Table 6).

## 4. Discussion

Periodontal infections are polymicrobial, and red complex and orange complex bacteria are frequently considered major periodontal pathogens [7]. Open-ended molecular approaches have been able to identify previously unidentified novel pathogens to be associated with periodontitis. The current study investigated the presence of *F. alocis* and *D. pneumosintes* in subgingival dental plaque in healthy gums and periodontitis and additionally their association with red complex bacteria in moderate to severe periodontitis.

This study demonstrated that both *F. alocis* and *D. pneumosintes* were detected at a significantly higher percentage in periodontitis subjects compared to healthy subjects. *F. alocis* and *D. pneumosintes* did not show a significant association with both *P. gingivalis* and *T. denticola* of the red complex bacteria. However, only *D. pneumosintes* demonstrated a significant association with *T. forsythia* in the P group. The detection of *F. alocis* and *D. pneumosintes* was significant at a higher CAL.

The demographic parameters were found to be comparable between the groups and the baseline clinical characteristics of the H and P groups were significantly different (*p* < 0.05). The P group demonstrated a higher occurrence of *F. alocis* compared to the H group (*p* < 0.05). This finding of our study is similar to those of previous studies that found periodontitis had an increased frequency and a higher number of *F. alocis* compared to healthy sites [38,39,40,41,42,43,44,45]. In 2021, Neelakandan et al. reported higher average counts of *F. alocis* in chronic periodontitis cases compared to healthy controls [46]. The evidence from an association and elimination study found a strong evidence of *F. alocis* being associated with periodontitis [9]. Similarly, another Brazilian study found *F. alocis* to be significantly increased in advanced periodontitis patients [29] (26). In our study, *D. pneumosintes* was detected more frequently in P subjects than in H subjects (*p*-value < 0.001). The results of our study are aligned with evidence from previous investigations of *D. pneumosintes* being associated with the oral microbiota of young adults and with advanced periodontal destruction [23,24,25]. A study by Ayala Herrera et al., in 2019, found that both *F. alocis* and *D. pneumosintes* were detected at higher frequencies (80% and 66.66%, respectively) in the Mexican population [47]. Ferraro et al. [22,48], in their study, found that periodontitis patients had a significantly greater mean prevalence of *D. pneumosintes* (62.1%) than periodontally healthy individuals (43.5%). In a recent study, *D. pneumosintes* was significantly more prevalent in periodontitis than in healthy individuals [29]. Nishiyama et al. did not detect *D. pneumosintes* in healthy samples but found this organism in 45.8% of the periodontitis samples [49]. In contrast, the association and elimination study using genomics demonstrated no specific association of *D. pneumosintes* with periodontitis [9]. This variation in the association of *D. pneumosintes* in different studies could also indicate that its prevalence varies among different populations. There is a need for more studies among these different populations to be able to determine the prevalence and role of *D. pneumosintes* in periodontitis.

The high incidence of *F. alocis* and *D. pneumosintes* in periodontitis patients, as seen in this study, could be because of their virulence traits. The unique virulence attributes of *F. alocis* include its tolerance to oxidative stress and its participation in periodontal biofilms that secrete different proteases to activate the host response. This host immune response then leads to the production of inflammatory mediators, like IL-1β, IL-6, and TNF-α, causing chronic inflammation and working synergistically with other periodontal pathogens [19,50]. On the other hand, *D. pneumosintes* is known to colonize both healthy sites and periodontitis sites, which along with its ability to interact with other organisms and known virulence factors can explain its high prevalence [22,27,28].

In the present study, an association of *F. alocis* with red complex bacteria in periodontally healthy and periodontitis subjects showed no significant difference. In a previous publication by our group, we found that *F. alocis* was positively correlated with *T. forsythia* in type 2 diabetes mellitus subjects with and without periodontitis [51]. A study by Chen et al. demonstrated that *F. alocis* was strongly correlated with *P. gingivalis*, *T. forsythia*, and five other bacteria in periodontitis subjects [52]. The results of the current study do not align with those of the previous studies, and this difference could be attributed to differences in techniques used to collect or process samples, periodontal disease status, and the population studied.

The study noted that both *D. pneumosintes* bacteria and *T. Forsythia* were detected more frequently in periodontitis. This finding is of great interest since, previously, *T. forsythia* is known to co-exist with *Fusobacterium nucleatum* [53], and this allows *T. forsythia* to obtain external N-acetylmuramic acid or other by-products from *Fusobacterium nucleatum* [54]. According to Ghayoumi et al. [55], *T. forsythia* may acquire growth factors from *D. pneumosintes*, or vice versa. Additionally, they outlined that some other organisms may provide growth factors to both *T. forsythia* and *D. pneumosintes*.

Among the clinical parameters, a higher clinical attachment loss has been associated with a greater severity of periodontal disease [1]. This study observed a significant association between clinical attachment loss and the presence of *F. alocis* and *D. pneumosintes*. The results of the study confirm the data from previous studies that have reported an increased prevalence of these bacteria with increased probing depths, higher clinical attachment loss, and increased disease severity [22,25,29,46,55,56,57]. The *F. alocis* genotype, which has an enhanced virulence, is found to be significantly associated with increased probing depths and progressive attachment loss [56,58]. The association of *D. pneumosintes* with periodontal inflammation and its probable interaction with the herpes virus have been associated with alveolar bone loss and attachment loss [22,28].

One main limitation of the current study was the small sample size, and therefore, caution must be exercised when interpreting the results. Secondly, a quantitative assessment of *F. alocis* and *D. pneumosintes* was not conducted because this investigation employed conventional PCR, which is a study limitation. The conventional PCR falls short because it cannot provide quantitative data. It would be intriguing to investigate how these bacteria’s different virulence factors contribute to the onset of inflammatory responses and their interaction with other microbial species in the oral ecological niche.

In conclusion, this study demonstrates that both *F. alocis* and *D. pneumosintes* are detected at a significantly higher percentage in periodontitis subjects compared to healthy subjects. Both *F. alocis* and *D. pneumosintes* had a higher frequency of being detected in sites with increased clinical attachment loss (moderate to severe periodontal disease). It was also evident that both *F. alocis* and *D. pneumosintes* can be present independently of other putative periodontal pathogens. Further studies are required to elucidate the role of these periodontal pathogens in the etiopathogenesis of periodontitis. There are limited data about the presence of these novel species in peri-implantitis, and further studies are required to fill this gap.

## Figures and Tables

**Table 1:** Primer sequences used in the study with their specific amplification length in base pairs.

Target	Primer Sequence (5′-3′) ^a^	Amplification Length (bp)
*Filifactor alocis*	CAGGTGGTTTAACAAGTTAGTGG CTAAGTTGTCCTTAGCTGTCTCG	594 [36]
*Dialister pneumosintes*	TTCTAAGCATCGCATGGTGC GATTTCGCTTCTCTTTGTTG	1105 [36]
*Porphyromonas gingivalis*	AGGCAGCTTGCCATACTGCG ACTGTTAGCAACTACCGATGT	404 [37]
*Treponema denticola*	TAATACCGAATGTGCTCATTTACAT TCAAAGAAGCATTCCCTCTTCTTCTTA	316 [37]
*Tannerella forsythia*	GCGTATGTAACCTGCCCGCA TGCTTCAGTGTCAGTTATACCT	641 [37]

**Table 2:** Comparison of the demographic and clinical parameters in the healthy and periodontitis groups.

Parameter	H Group	P Group	*p*-Value
Gender (Male/Female)	12/18	13/17	0.7934 *
Age (years)	44.03 ± 10.11	42.07 ± 10.60	0.465 ^#^
GI	0.100 ± 0.043	2.09 ± 0.188	<0.0001 ^γ^
PI	0.094 ± 0.040	2.23 ± 0.193	<0.0001 ^γ^
BI	3.098 ± 2.191	93.30 ± 6.517	<0.0001 ^γ^
PD	1.465 ± 0.333	5.361 ± 0.252	<0.0001 ^γ^
CAL	0.000 ± 0.000	5.014 ± 0.566	<0.0001 ^γ^

* Chi-squared test; ^#^ *T*-test; ^γ^ Mann–Whitney U test. GI: gingival index, PI: plaque index, BI: bleeding index, PD: probing depth, CAL: clinical attachment loss, H group: healthy group, P group: periodontitis group.

**Table 3:** Comparison of the healthy and periodontitis groups for the occurrence of *F. alocis*, *D. pneumosintes*, and red complex bacteria by Fisher’s exact test.

Organisms	Negative/ Positive	H Group	P Group	Total	Fisher’s Exact Test *p*-Value
***F. alocis***	Negative	24 (80%)	14 (46.7)	38 (63.3%)	0.015 *
	Positive	6 (20%)	16 (53.3%)	22 (36.7%)	
***D. pneumosintes***	Negative	24 (80%)	10 (33.33%)	34 (56.67)	0.0006 *
	Positive	6 (20%)	20 (66.67%)	26 (43.33)	
***P. gingivalis***	Negative	24 (80%)	17 (56.67)	41 (68.33)	0.09
	Positive	6 (20%)	13 (43.33)	19 (31.67)	
***T. denticola***	Negative	24 (80%)	14 (46.7)	38 (63.3%)	0.015 *
	Positive	6 (20%)	16 (53.3%)	22 (36.7%)	
***T. forsythia***	Negative	22 (73.33)	21 (70.00)	43 (71.67)	1.0
	Positive	8 (26.67)	9 (30.00)	17 (28.33)	

H group: healthy group, P group: periodontitis group. Statistical analysis is based on the comparison of the positive sites of the H and P groups only. * has been added to only the signifcant data sets.

**Table 4:** Correlation of *F. alocis* with red complex bacteria in the healthy and periodontitis groups by Fisher’s exact test.

Group	Red Complex Bacteria		*F. alocis*		Total	Fisher’s Exact Test *p*-Value
			Negative	Positive		
**H group (N = 30)**	***P. gingivalis***	Negative	19 (79.2%)	5 (20.8%)	24 (100.0%)	1.0
		Positive	5 (83.3%)	1 (16.7%)	6 (100.0%)	
	***T. denticola***	Negative	20 (83.3%)	4 (16.7%)	24 (100.0%	0.5705
		Positive	4 (66.7%)	2 (33.3%)	6 (100.0%)	
	***T. forsythia***	Negative	16 (72.7%)	6 (27.3%)	22 (100.0%)	0.1550
		Positive	8 (100.0%)	0 (0%)	8 (100.0%)	
**P group (N = 30)**	***P. gingivalis***	Negative	6 (35.3%)	11 (64.7%)	17 (100.0%)	0.2685
		Positive	8 (61.5%)	5 (38.5%)	13 (100.0%)	
	***T. denticola***	Negative	9 (64.3%)	5 (35.7%)	14 (100.0%)	0.1414
		Positive	5(31.3%)	11 (68.8%)	16 (100.0%)	
	***T. forsythia***	Negative	12 (57.1%)	9 (42.9%)	21(100.0%)	0.1184
		Positive	2 (22.2%)	7 (77.8%)	9 (100.0%)	

H group: healthy group, P group: periodontitis group. Statistical analysis is based on the comparison of the positive sites of the H and P groups only.

**Table 5:** Correlation of *D. pneumosintes* with red complex bacteria in the healthy and periodontitis groups by Fisher’s exact test.

Group	Red Complex Bacteria		*D. pneumosintes*		Total	Fisher’s Exact Test *p*-Value
			Negative	Positive		
**H group (N = 30)**	***P. gingivalis***	Negative	20 (83.33%)	4 (16.67%)	24 (100.0%)	0.5705
		Positive	4 (66.67%)	2 (33.33%)	6 (100.0%)	
	***T. denticola***	Negative	19 (79.17%)	5 (20.83%)	24 (100.0%	1.0000
		Positive	5 (83.33%)	1 (16.67%)	6 (100.0%)	
	***T. forsythia***	Negative	17 (77.27%)	5 (22.73%)	22 (100.0%)	1.0000
		Positive	7 (87.50%)	1 (12.50%)	8 (100.0%)	
**P group (N = 30)**	***P. gingivalis***	Negative	4 (23.53%)	13 (76.47%)	17 (100.0%)	0.2553
		Positive	6 (46.15%)	7 (53.85%)	13 (100.0%)	
	***T. denticola***	Negative	6 (42.86%)	8 (57.14%)	14 (100.0%)	0.4421
		Positive	4 (25.00%)	12 (75.00%)	16 (100.0%)	
	***T. forsythia***	Negative	10 (47.62%)	11 (52.38%)	21(100.0%)	0.0134 *
		Positive	0 (0.00%)	9 (100%)	9 (100.0%)	

H group: healthy group, P group: periodontitis group. Statistical analysis is based on the comparison of the positive sites of the H and P groups only. * has been added to only the signifcant data sets.

**Table 6:** Comparison of clinical attachment loss with *F. alocis*- and *D. pneumosintes*-positive and -negative cases by Fisher’s exact test.

Clinical Attachment Loss	*F. alocis*		Fisher’s Exact Test *p*-Value	*D. pneumosintes*		Fisher’s Exact Test *p*-Value
	Negative	Positive		Negative	Positive	
**<3 mm**	24 (80.0%)	6 (20.0%)	0.024 *	24 (80%)	6 (20%)	0.0007 *
**3–5 mm**	8 (44.4%)	10 (55.6%)		5 (27.77%)	13 (72.23%)	
**>5 mm**	6 (50.0%)	6 (50.0%)		5 (41.66%)	7 (58.34%)	

* has been added to only the signifcant data sets.

## Data Availability

The data presented in this study are available upon request from the corresponding author.

## Supplementary Materials

The following supporting information can be downloaded at: https://www.mdpi.com/article/10.3390/dj12040105/s1. Table S1: STROBE Statement—Checklist of items that should be included in reports of cross-sectional studies.

## Abbreviations

DNA	Deoxyribonucleic acid
H group	Healthy group
P group	Periodontitis group
CAL	Clinical attachment loss
PD	Probing depth
BOP	Bleeding on probing
PI	Plaque index
GI	Gingival index
BI	Bleeding index
PCR	Polymerase chain reaction
TE	Tris-EDTA buffer
16SrRNA	16S ribosomal ribonucleic acid
IL-1β	Interleukin-1β
IL-6	Interleukin-6
TNF-α	Tumour necrosis factor alpha

## References

[1] Papapanou P.N., Sanz M., Buduneli N., Dietrich T., Feres M., Fine D.H., Flemmig T.F., Garcia R., Giannobile W.V., Graziani F. (2018). Periodontitis: Consensus Report of Workgroup 2 of the 2017 World Workshop on the Classification of Periodontal and Peri-Implant Diseases and Conditions. J. Clin. Periodontol..

[2] Abusleme L., Hoare A., Hong B.-Y., Diaz P.I. (2021). Microbial Signatures of Health, Gingivitis, and Periodontitis. Periodontol. 2000.

[3] Naginyte M., Do T., Meade J., Devine D.A., Marsh P.D. (2019). Enrichment of Periodontal Pathogens from the Biofilms of Healthy Adults. Sci. Rep..

[4] Bašić K., Peroš K., Bošnjak Z., Šutej I. (2021). Subgingival Microbiota Profile in Association with Cigarette Smoking in Young Adults: A Cross-Sectional Study. Dent. J..

[5] Bernardi S., Qorri E., Botticelli G., Scarano A., Marzo G., Gatto R., Greco Lucchina A., Mortellaro C., Lupi E., Rastelli C. (2023). Use of Electrical Field for Biofilm Implant Removal. Eur. Rev. Med. Pharmacol. Sci..

[6] Franco R., Rosa A., Lupi E., Capogreco M. (2023). The Influence of Dental Implant Roughness on Biofilm Formation: A Comprehensive Strategy. Dent. Hypotheses.

[7] Socransky S.S., Haffajee A.D., Cugini M.A., Smith C., Kent R.L. (1998). Microbial Complexes in Subgingival Plaque. J. Clin. Periodontol..

[8] Antezack A., Etchecopar-Etchart D., La Scola B., Monnet-Corti V. (2023). New Putative Periodontopathogens and Periodontal Health-Associated Species: A Systematic Review and Meta-Analysis. J. Periodontal Res..

[9] Veras E.L., Castro Dos Santos N., Souza J.G.S., Figueiredo L.C., Retamal-Valdes B., Barão V.A.R., Shibli J., Bertolini M., Faveri M., Teles F. (2023). Newly Identified Pathogens in Periodontitis: Evidence from an Association and an Elimination Study. J. Oral Microbiol..

[10] Aas J.A., Paster B.J., Stokes L.N., Olsen I., Dewhirst F.E. (2005). Defining the Normal Bacterial Flora of the Oral Cavity. J. Clin. Microbiol..

[11] Pérez-Chaparro P.J., Gonçalves C., Figueiredo L.C., Faveri M., Lobão E., Tamashiro N., Duarte P., Feres M. (2014). Newly Identified Pathogens Associated with Periodontitis: A Systematic Review. J. Dent. Res..

[12] Hiranmayi K.V., Sirisha K., Ramoji Rao M.V., Sudhakar P. (2017). Novel Pathogens in Periodontal Microbiology. J. Pharm. Bioallied Sci..

[13] Kim H.Y., Song M.-K., Gho Y.S., Kim H.-H., Choi B.-K. (2021). Extracellular Vesicles Derived from the Periodontal Pathogen Filifactor Alocis Induce Systemic Bone Loss through Toll-like Receptor 2. J. Extracell. Vesicles.

[14] Yoo H.-J., Lee S.-H. (2022). Virulence of Filifactor Alocis Lipoteichoic Acid on Human Gingival Fibroblast. Arch. Oral Biol..

[15] Aruni A.W., Zhang K., Dou Y., Fletcher H. (2014). Proteome Analysis of Coinfection of Epithelial Cells with Filifactor Alocis and Porphyromonas Gingivalis Shows Modulation of Pathogen and Host Regulatory Pathways. Infect. Immun..

[16] Aruni A.W., Roy F., Fletcher H.M. (2011). Filifactor Alocis Has Virulence Attributes That Can Enhance Its Persistence under Oxidative Stress Conditions and Mediate Invasion of Epithelial Cells by Porphyromonas Gingivalis. Infect. Immun..

[17] Moffatt C.E., Whitmore S.E., Griffen A.L., Leys E.J., Lamont R.J. (2011). Filifactor Alocis Interactions with Gingival Epithelial Cells. Mol. Oral Microbiol..

[18] Dahlén G., Claesson R., Aberg C.H., Haubek D., Johansson A., Kwamin F. (2014). Subgingival Bacteria in Ghanaian Adolescents with or without Progression of Attachment Loss. J. Oral Microbiol..

[19] Schlafer S., Riep B., Griffen A.L., Petrich A., Hübner J., Berning M., Friedmann A., Göbel U.B., Moter A. (2010). Filifactor Alocis--Involvement in Periodontal Biofilms. BMC Microbiol..

[20] Ozuna H., Snider I., Belibasakis G.N., Oscarsson J., Johansson A., Uriarte S.M. (2022). Aggregatibacter Actinomycetemcomitans and Filifactor Alocis: Two Exotoxin-Producing Oral Pathogens. Front. Oral Health.

[21] Olitsky P.K., Gates F.L. (1921). Experimental Studies of the Nasopharyngeal Secretions From Influenza Patients: I. Transmission Experiments With Nasopharyngeal Washings. J. Exp. Med..

[22] Ferraro C.T.L., Gornic C., Barbosa A.S., Peixoto R.J.M., Colombo A.P.V. (2007). Detection of Dialister Pneumosintes in the Subgingival Biofilm of Subjects with Periodontal Disease. Anaerobe.

[23] Doan N., Contreras A., Flynn J., Slots J., Chen C. (2000). Molecular Identification of Dialister Pneumosintes in Subgingival Plaque of Humans. J. Clin. Microbiol..

[24] Contreras A., Doan N., Chen C., Rusitanonta T., Flynn M.J., Slots J. (2000). Importance of Dialister Pneumosintes in Human Periodontitis. Oral Microbiol. Immunol..

[25] Zhao Y., Ye Q., Feng Y., Chen Y., Tan L., Ouyang Z., Zhao J., Hu J., Chen N., Su X. (2022). Prevotella Genus and Its Related NOD-like Receptor Signaling Pathway in Young Males with Stage III Periodontitis. Front. Microbiol..

[26] Drago L., Vassena C., Saibene A.M., Del Fabbro M., Felisati G. (2013). A Case of Coinfection in a Chronic Maxillary Sinusitis of Odontogenic Origin: Identification of Dialister Pneumosintes. J. Endod..

[27] Jumas-Bilak E., Jean-Pierre H., Carlier J.-P., Teyssier C., Bernard K., Gay B., Campos J., Morio F., Marchandin H. (2005). *Dialister micraerophilus* sp. nov. and *Dialister propionicifaciens* sp. nov., Isolated from Human Clinical Samples. Int. J. Syst. Evol. Microbiol..

[28] Slots J., Sugar C., Kamma J.J. (2002). Cytomegalovirus Periodontal Presence Is Associated with Subgingival Dialister Pneumosintes and Alveolar Bone Loss. Oral Microbiol. Immunol..

[29] Araújo L.L., Lourenço T.G.B., Colombo A.P.V. (2023). Periodontal Disease Severity Is Associated to Pathogenic Consortia Comprising Putative and Candidate Periodontal Pathogens. J. Appl. Oral Sci..

[30] Faul F., Erdfelder E., Buchner A., Lang A.-G. (2009). Statistical Power Analyses Using G*Power 3.1: Tests for Correlation and Regression Analyses. Behav. Res. Methods.

[31] von Elm E., Altman D.G., Egger M., Pocock S.J., Gøtzsche P.C., Vandenbroucke J.P. (2007). Strengthening the Reporting of Observational Studies in Epidemiology (STROBE) Statement: Guidelines for Reporting Observational Studies. BMJ.

[32] Silness J., Loe H. (1964). Periodontal Disease In Pregnancy. Ii. Correlation Between Oral Hygiene and Periodontal Condtion. Acta Odontol. Scand..

[33] Loe H., Silness J. (1963). Periodontal Disease In Pregnancy. I. Prevalence and Severity. Acta Odontol. Scand..

[34] Carter H.G., Barnes G.P. (1974). The Gingival Bleeding Index. J. Periodontol..

[35] Kugaji M.S., Bhat K.G., Joshi V.M., Pujar M., Mavani P.T. (2017). Simplified Method of Detection of *Dialister* Invisus and *Olsenella Uli* in Oral Cavity Samples by Polymerase Chain Reaction. J. Adv. Oral Res..

[36] Siqueira J.F., Rôças I.N. (2004). Simultaneous Detection of Dialister Pneumosintes and Filifactor Alocis in Endodontic Infections by 16S RDNA-Directed Multiplex PCR. J. Endod..

[37] Ashimoto A., Chen C., Bakker I., Slots J. (1996). Polymerase Chain Reaction Detection of 8 Putative Periodontal Pathogens in Subgingival Plaque of Gingivitis and Advanced Periodontitis Lesions. Oral Microbiol. Immunol..

[38] Kumar P.S., Griffen A.L., Moeschberger M.L., Leys E.J. (2005). Identification of Candidate Periodontal Pathogens and Beneficial Species by Quantitative 16S Clonal Analysis. J. Clin. Microbiol..

[39] Kumar P.S., Leys E.J., Bryk J.M., Martinez F.J., Moeschberger M.L., Griffen A.L. (2006). Changes in Periodontal Health Status Are Associated with Bacterial Community Shifts as Assessed by Quantitative 16S Cloning and Sequencing. J. Clin. Microbiol..

[40] Dahlén G., Leonhardt A. (2006). A New Checkerboard Panel for Testing Bacterial Markers in Periodontal Disease. Oral Microbiol. Immunol..

[41] Colombo A.P.V., Boches S.K., Cotton S.L., Goodson J.M., Kent R., Haffajee A.D., Socransky S.S., Hasturk H., Van Dyke T.E., Dewhirst F. (2009). Comparisons of Subgingival Microbial Profiles of Refractory Periodontitis, Severe Periodontitis, and Periodontal Health Using the Human Oral Microbe Identification Microarray. J. Periodontol..

[42] Lindholm M., Claesson R., Löf H., Chiang H.-M., Oscarsson J., Johansson A., Åberg C.H. (2023). Radiographic and Clinical Signs of Periodontitis and Associated Bacterial Species in a Swedish Adolescent Population. J. Periodontol..

[43] Ikeda E., Shiba T., Ikeda Y., Suda W., Nakasato A., Takeuchi Y., Azuma M., Hattori M., Izumi Y. (2020). Japanese Subgingival Microbiota in Health vs. Disease and Their Roles in Predicted Functions Associated with Periodontitis. Odontology.

[44] Ko Y., Lee E.-M., Park J.C., Gu M.B., Bak S., Ji S. (2020). Salivary Microbiota in Periodontal Health and Disease and Their Changes Following Nonsurgical Periodontal Treatment. J. Periodontal Implant Sci..

[45] Ji S., Kook J.-K., Park S.-N., Lim Y.K., Choi G.H., Jung J.-S. (2023). Characteristics of the Salivary Microbiota in Periodontal Diseases and Potential Roles of Individual Bacterial Species to Predict the Severity of Periodontal Disease. Microbiol. Spectr..

[46] Neelakandan A., Potluri R., Yadalam P.K., Chakraborty P., Saravanan A.V., Arunraj R. (2021). The Varied Proportion of Filifactor Alocis in Periodontal Health and Disease in the South Indian Subpopulation. Contemp. Clin. Dent..

[47] Ayala Herrera J.L., Apreza Patrón L., Martínez Martínez R.E., Domínguez Pérez R.A., Abud Mendoza C., Hernández Castro B. (2019). Filifactor Alocis and Dialister Pneumosintes in a Mexican Population Affected by Periodontitis and Rheumatoid Arthritis: An Exploratory Study. Microbiol. Immunol..

[48] Nishiyama S.A.B., Nakano V., Velásquez-Melendez G., Avila-Campos M.J. (2008). Occurrence of Herpes Simplex Virus 1 and Three Periodontal Bacteria in Patients with Chronic Periodontitis and Necrotic Pulp. Can. J. Microbiol..

[49] Aruni A.W., Mishra A., Dou Y., Chioma O., Hamilton B.N., Fletcher H.M. (2015). Filifactor Alocis—A New Emerging Periodontal Pathogen. Microbes Infect..

[50] Shaikh H.F.M., Oswal P.U., Kugaji M.S., Katti S.S., Bhat K.G., Joshi V.M. (2023). Co-Occurrence of Filifactor Alocis with Red Complex Bacteria in Type 2 Diabetes Mellitus Subjects with and without Chronic Periodontitis: A Pilot Study. Int. J. Transl. Med..

[51] Chen H., Liu Y., Zhang M., Wang G., Qi Z., Bridgewater L., Zhao L., Tang Z., Pang X. (2015). A Filifactor Alocis-Centered Co-Occurrence Group Associates with Periodontitis across Different Oral Habitats. Sci. Rep..

[52] Wyss C. (1989). Dependence of Proliferation of Bacteroides Forsythus on Exogenous N-Acetylmuramic Acid. Infect. Immun..

[53] Haffajee A.D., Socransky S.S. (1994). Microbial Etiological Agents of Destructive Periodontal Diseases. Periodontol. 2000.

[54] Ghayoumi N., Chen C., Slots J. (2002). Dialister Pneumosintes, a New Putative Periodontal Pathogen. J. Periodontal Res..

[55] Oliveira R.R.D.S., Fermiano D., Feres M., Figueiredo L.C., Teles F.R.F., Soares G.M.S., Faveri M. (2016). Levels of Candidate Periodontal Pathogens in Subgingival Biofilm. J. Dent. Res..

[56] Razooqi Z., Höglund Åberg C., Kwamin F., Claesson R., Haubek D., Oscarsson J., Johansson A. (2022). Aggregatibacter Actinomycetemcomitans and Filifactor Alocis as Associated with Periodontal Attachment Loss in a Cohort of Ghanaian Adolescents. Microorganisms.

[57] Gonçalves C., Soares G.M.S., Faveri M., Pérez-Chaparro P.J., Lobão E., Figueiredo L.C., Baccelli G.T., Feres M. (2016). Association of Three Putative Periodontal Pathogens with Chronic Periodontitis in Brazilian Subjects. J. Appl. Oral Sci..

[58] Feres M., Retamal-Valdes B., Gonçalves C., Cristina Figueiredo L., Teles F. (2021). Did Omics Change Periodontal Therapy?. Periodontol. 2000.

